# Supplementary material for: Patient satisfaction with healthcare services among health insurance program beneficiaries in Nepal: A cross-sectional study
Source: PLoS One. 2025 Nov 12;20(11):e0334352. doi: 10.1371/journal.pone.0334352 (PMC12611131; doi:10.1371/journal.pone.0334352)
Supplement: S1 Table — (DOCX) [file pone.0334352.s001.docx]

**S1 Table: List of Study Sites**

| **Province** | **S.N.** | **District Name** | **Name of Health Facility** | **Category** |
| --- | --- | --- | --- | --- |
| Bagmati Province | 1 | Kathmandu | Bir Hospital | Public Hospital |
|  | 2 | Kathmandu | Sahid Gangalal National Heart Center | Public Hospital |
|  | 3 | Chitwan | College of Medical Sciences | Community/Pvt Hospitals/Academic |
|  | 4 | Rasuwa | District Hospital, Rasuwa | Public Hospital |
|  | 5 | Chitwan | Bakulahar Ratnagar Hospital | PHCC |
|  | 6 | Makwanpur | Hetauda Hospital | Public Hospital |
|  | 7 | Bhaktapur | Surya Binayak Nagar Aspatal | PHCC |
|  | 8 | Sindhupalchowk | Barahbise PHCC | PHCC |
|  | 9 | Kathmandu | Chalnakhel Hospital | PHCC |
|  | 10 | Kathmandu | Tilganaga Institue of Opthalmology | Public Hospital |
| Madesh Province | 11 | Parsa | Narayani Hospital | Public Hospital |
|  | 12 | Sarlahi | Malangwa District Hospital, Sarlahi | Public Hospital |
|  | 13 | Sarlahi | Lalbandi PHC | PHCC |
|  | 14 | Mahottari | Gaushala PHC | PHCC |
|  | 15 | Mahottari | Bardibas Nagar Aspatal | PHCC |
|  | 16 | Saptari | Chinnamasta Educational Academy & Medical College Pvt.Ltd | Community/Pvt Hospitals/Academic |
| Karnali Province | 17 | Surkhet | Province Hospital | Public Hospital |
|  | 18 | Surkhet | Surkhet Eye Hospital | Public Hospital |
|  | 19 | Jajarkot | Dalli Municipal Hospital | Public Hospital |
|  | 20 | West Rukum | Chaurjahari Mission Hospital | Community/Pvt Hospitals/Academic |
|  | 21 | Kalikot | Kalikot District Hospital | Public Hospital |
|  | 22 | Surkhet | Mehelkuna Hospital | Public Hospital |

PHCC= Primary Health Care Centre
