# Supplementary material for: Patient satisfaction with healthcare services among health insurance program beneficiaries in Nepal: A cross-sectional study
Source: PLoS One. 2025 Nov 12;20(11):e0334352. doi: 10.1371/journal.pone.0334352 (PMC12611131; doi:10.1371/journal.pone.0334352)
Supplement: S2 Table — (DOCX) [file pone.0334352.s002.docx]

**S2 Table: Scoring system of Patient Satisfaction Questionnaire III**

**Instructions for Scoring the PSQ-III**

The PSQ-III yields separate scores for each of seven different subscales: General Satisfaction (Items 3 and 17); Technical Quality (Items 2, 4, 6, and 14); Interpersonal Manner (Items 10 and 11); Communication (Items 1 and 13); Financial Aspects (Items 5 and 7); Time Spent with Doctor (Items 12 and 15); Accessibility and Convenience (Items 8, 9, 16, and 18).

**Scoring of the 18 Items**

| **Item Numbers** | **Original Response Value** | **Scored Value** |
| --- | --- | --- |
| 1,2,3,5,6,8,11,15,18 | 1 | 1 |
|  | 2 | 2 |
|  | 3 | 3 |
|  | 4 | 4 |
|  | 5 | 5 |
| 4,7,9,10,12,13,14,16,17 | 1 | 5 |
|  | 2 | 4 |
|  | 3 | 3 |
|  | 4 | 2 |
|  | 5 | 1 |

**Creating Scale Scores**

| **Scale** | **Average These Items** |
| --- | --- |
| General Satisfaction | 3, 17 |
| Technical Quality | 2, 4, 6, 17 |
| Interpersonal Manner | 10, 11 |
| Communication | 1, 13 |
| Financial Aspects | 5, 7 |
| Time spent with Doctor | 12, 15 |
| Accessibility and Convenience | 8, 9, 16, 18 |
