# Supplementary material for: Patient satisfaction with healthcare services among health insurance program beneficiaries in Nepal: A cross-sectional study
Source: PLoS One. 2025 Nov 12;20(11):e0334352. doi: 10.1371/journal.pone.0334352 (PMC12611131; doi:10.1371/journal.pone.0334352)
Supplement: Attachment — Submitted filename: Response to Reviewers.docx [file pone.0334352.s005.docx]

**Response to Reviewer’s Comments**

**We would like to thank all the reviewers for their time and effort to read and comment on our paper. Their comments are constructive and the paper has benefitted from it. Below we present the point-by-point response to the reviewer’s comments.**

**REVIEWER 1**

| **Comments** | **Responses** |
| --- | --- |
| 1. Sample Size Justification Comment: The Methods state that “A total of 468 insured patients … were included” but no a priori power or sample-size calculation is provided to justify this number for the planned multi-ordinal logistic regressions (pp. 13–14, L126–131).  Recommendation: Please include a power analysis—specifying expected effect sizes, number of predictors, desired power (e.g., 80 %), and α-level—to confirm that n = 468 is sufficient to detect meaningful associations. | Thank you for the comment.  We have added the following statement in the sampling section:  ***We calculated the sample size using the prevalence formula: n= Z^2^(p(1-p))/d^2^keeping prevalence at 50%. This formula gave us the minimum sample size of 385. With addition of a 20% non-response rate, the sample was calculated as: 462. The data was collected from a total sample of 468 at the end of data collection period.*** |
| 2. Handling of Likert-Scale Data Comment: Satisfaction domain scores are calculated by averaging 18 PSQ-III items on a 1–5 Likert scale (pp. 20–21, L223–228). Treating ordinal data as continuous may bias estimates if scale assumptions are violated.  Recommendation: Report Cronbach’s α for each domain in the full sample (not only the pre-test) to confirm internal consistency. Conduct sensitivity analyses using ordinal methods (e.g., factor analysis on the ordinal items or GEE for ordinal outcomes) to verify that mean-based scores yield similar results. | Thank you for this valuable suggestion.  Cronbach's alpha was calculated as **0.8037**. We have reported it in the method section as well.  We would like to clarify that our analysis already treats each of the 7 domains as ordinal outcomes, following the standard structure and scoring guidance of the PSQ-III tool (https://www.rand.org/health-care/surveys_tools/psq.html#:~:text=The%20PSQ%2DIII%20is%20a,III%20is%20also%20available%20below.)  In our analysis, we had used ordinal logistic regression (ologit) to model the relationship between each domain and relevant predictors. In the revised version, we have presented our findings by employing the Generalized ordered logistic regression.  The methods section has been revised accordingly. |
| 3. Choice of Multi-Ordinal Logistic Regression Comment: The manuscript uses multi-ordinal logistic regression for three outcome categories (“dissatisfied,” “neutral,” “satisfied”) but does not report testing the proportional-odds assumption (pp. 8–9, L37–42).  Recommendation: Provide diagnostics (e.g., Brant test) for the proportional-odds assumption. If violated, consider partial proportional-odds or multinomial logistic models to avoid biased odds ratios. | We acknowledge your important point regarding the proportional odds assumption in ordinal logistic regression.  We have employed the Generalized Ordered Logistic Regression (gologit2) with the autofit option, for the partial proportional odds.  The results of this model are now reported in the revised manuscript (Table 4). |
| 4. Model Selection and Overfitting Comment: Variable selection used stepwise forward selection based on AIC (pp. 15–16, L25–28). Stepwise methods can inflate Type I error and risk overfitting when many predictors are considered.  Recommendation: Report the number of parameters relative to events per parameter in each domain model. Consider penalized approaches (e.g., LASSO) or cross-validation to assess model stability. Present full (“all-covariate”) models alongside the reduced models to demonstrate robustness of selected predictors. | **Thank you for this important comment.** We recognize the limitations of stepwise selection methods, including the potential for overfitting and inflated Type I error, particularly when numerous predictors are evaluated.  We have revised our modality. We selected the best-fit model using stepwise selection method. Following this, we applied the Generalized Ordered logistic Regression with the autofit option to account for the partial proportional odds assumption. We have revised the analysis section likewise.  We have presented the reduced model in Table 4 and also presented the full model as supplemental file 4. |
| 5. Presentation of Regression Results Comment: Table 4 displays only statistically significant predictors, obscuring potentially important confounders (pp. 24–25, L249–252). Recommendation: Publish complete regression tables—including non-significant covariates—with AORs, 95 % CIs, and p-values for all variables to enhance transparency and allow readers to assess confounding. | Thank you for your feedback.  We have reported the full model table as Supplemental File 4. |
| 6. Adjustment for Multiple Comparisons Comment: Conducting seven separate domain-specific regressions increases the risk of spurious findings (pp. 24–25, Table 4). Recommendation: Apply a multiple-testing correction (e.g., Bonferroni or Benjamini–Hochberg) or explicitly state that analyses are exploratory, to contextualize p-values—particularly those near α = 0.05. | We have reported the Bonferroni corrected P-value in Table 4.  Also, we have stated that the analysis is exploratory. (In method section) |

**Reviewer 2**

We greatly appreciate the effort in your study assessing patient satisfaction under Nepal’s NHIP. To further enhance the clarity and rigor of your manuscript, we suggest the following statistical and methodological clarifications and improvements:

| Comments | Responses |
| --- | --- |
| 1. Justification of Reference Categories in Regression Models In your ordinal logistic regression models, the choice of reference groups (e.g., "no formal education" for education level, "age below 20" for age, "agriculture" for occupation) appears arbitrary and lacks a clear rationale. Reference categories should ideally be selected based on: - Policy relevance (e.g., the majority or socially normative group), - Interpretation convenience (e.g., stable or meaningful baseline), - Statistical stability (e.g., groups with sufficient sample size).  Using the first-listed category by default can lead to unstable or inflated odds ratios, particularly when categories differ significantly in sample size or variance (e.g., age below 20 vs. above 60 years in age groups). We recommend re-examining the choice of reference groups and explicitly justifying them within the methods section. | **Thank you for this insightful observation.** We appreciate the importance of selecting reference categories thoughtfully to ensure both interpretability and statistical stability.  In the revised version, we have taken the reference groups based on the information from this article (<https://dhsprogram.com/pubs/pdf/WP199/WP199.pdf>).  We have referred to the groups with highest enrollment rates as reference groups. |
| 2. Incomplete Reporting of Multinomial Logistic Regression Results In Table 4, the regression results are presented selectively, often showing odds ratios only for one or two categories (e.g., only "60+ age group" without comparisons to other age groups). This selective presentation limits the reader's ability to: - Compare all levels within a categorical variable, - Understand the overall effect pattern of the variable, - Detect potential suppression or confounding effects. If space is limited, we recommend including full regression tables (with all categories) as supplementary material, and clearly stating whether the table includes only statistically significant. | Thank you for your feedback.  We have presented the results for all categories.  Also, the full table has been uploaded as the supplemental file 4. |
| 3. Lack of linkage between descriptive and inferential statistics Table 1 provides useful descriptive statistics on patients’ socioeconomic characteristics, but lacks inferential statistical analysis to assess whether the categorizations used are appropriate and whether there are significant differences across groups. When variables have three or more categories, statistical tests such as ANOVA or chi-square tests can be employed to compare satisfaction levels across categories. These analyses would help clarify the structure of associations between patient characteristics and satisfaction, and reinforce the rationale behind the regression model specifications. | The chi-square values of the variables for each domain are added as supplemental file. However, we did not rely on the chi-square values for model selection.  Methodologically, we conducted a stepwise selection method for choosing the best-fit model, followed by the Generalized ordered logistic regression with partial proportional odds model. |
| 4. Disconnection Between Analytical Findings and Conclusions The main findings of your regression models emphasize associations between patient satisfaction and socioeconomic characteristics (e.g., age, education, chronic illness). However, the conclusions emphasize only domain-level satisfaction (e.g., low satisfaction in financial and accessibility aspects) and call for broader health system strengthening—without referencing the regression findings. This weakens the internal logic of the study. We suggest aligning the conclusions more directly with your analytical results—for instance, noting that vulnerable groups (e.g., older adults, lower-educated, subsidized patients) were systematically less satisfied, and that targeted policy actions may be needed for these groups. | Thank you for your feedback.  We have now revised the conclusion with addition of information from the associated variables. |
