# Supplementary material for: Patient satisfaction with healthcare services among health insurance program beneficiaries in Nepal: A cross-sectional study
Source: PLoS One. 2025 Nov 12;20(11):e0334352. doi: 10.1371/journal.pone.0334352 (PMC12611131; doi:10.1371/journal.pone.0334352)
Supplement: Attachment — Submitted filename: Response_to_Reviewers_auresp_2.docx [file pone.0334352.s006.docx]

**Editor Comments**

***Please go through the minor comments from the reviewer. Also, I would like to request you to go through the grammatical/spell errors in the manuscripts (as in Line 123).***
 **Response:** We have carefully reviewed the entire manuscript and grammatical as well as spelling errors have been corrected to improve clarity and readability.

***You can also write in methods/ data analysis part - why you choose the reference value for bivariate/multivariate analysis.***

**Response:** Thank you for the comment. For the analysis, we conducted stepwise selection method for selecting the best-fit model in our analysis. This model was then assessed for partial proportional odds assumption using generalized ordered logistic regression.

We have included these details in our methods section as well.

**Reviewer’s comments**

***Please provide additional details on the generalized ordered logistic regression, including how the proportional odds assumption was tested and whether it was satisfied.***
 **Response:** Thank you for the comments. We have added the following statement in methods section for clarity to the readers.

*We assessed the proportional odds assumption for each variable included in the generalized ordered logistic regression. For variables that satisfied the proportional odds assumption, the threshold outcomes are presented as “All.” For variables that did not satisfy the assumption, separate adjusted odds ratios (AORs) are reported for each comparison (i.e., satisfied vs. dissatisfied and satisfied vs. neutral).*

***In Table 1, please add a note explaining the presence of missing data for variables where N is less than 468.***

**Response:** Thank you for the comment. We have added a note in the footnote of the Table.
